# Bidirectional Planar Flexible Snake‐Origami Batteries

**DOI:** 10.1002/advs.202101372

**Published:** 2021-08-27

**Authors:** Na Li, Haosen Chen, Shuangquan Yang, Heng Yang, Shuqiang Jiao, Wei‐Li Song

**Affiliations:** ^1^ Institute of Advanced Structure Technology Beijing Institute of Technology Beijing 100081 China; ^2^ Beijing Key Laboratory of Lightweight Multi‐Functional Composite Materials and Structures Beijing Institute of Technology Beijing 100081 P. R. China; ^3^ State Key Laboratory of Advanced Metallurgy University of Science and Technology Beijing Beijing 100083 P. R. China

**Keywords:** bidirectional, high energy density, planar flexible batteries, snake origami

## Abstract

With the rapid development of commercial flexible/wearable devices, flexible batteries have attracted great attention as optimal power sources. However, a combination of high energy density and excellent arbitrary deformation ability is still a critical challenge to satisfy practical applications. Inspired by rigid and soft features of chemical molecular structures, novel bidirectional flexible snake‐origami lithium‐ion batteries (LIBs) with both high energy density and favorable flexibility are designed and fabricated. The flexible snake‐origami battery consists of rigid and soft segments, where the former is designed as the energy unit and the latter served as the deformation unit. With the unique features from such design, the as‐fabricated battery with calculating all the components exhibits a record‐setting energy density of 357 Wh L^−1^ (133 Wh kg^−1^), compared with the cell‐scale flexible LIBs achieved from both academic and industry. Additionally, a design principle is established to verify the validity of utilizing rigid‐soft‐coupled structure for enduring various deformations, and the intrinsic relationship between battery structure, energy density, and flexibility can be confirmed. The results suggest that the design principle and performance of bidirectional flexible snake‐origami batteries will provide a new reliable strategy for achieving high energy flexible batteries for wearable devices.

## Graphical abstract

A novel bidirectional snake‐origami flexible battery of scalable planar structure is designed, inspired by rigid‐soft coupling features of chemical molecular structures, with two types of functional units, energy unit (rigid segments) and deformation unit (soft segments). The as‐fabricated batteries exhibit superior energy density of 357 Wh/L, along with robust energy storage performance even under mechanical deformation.

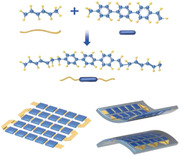

## 1 Introduction

With the rapid development of flexible/wearable devices in the areas such as medical, sensors, electronics and artificial skins, and flexible power sources that possess both high energy density and superior flexibility and durability are urgently required to provide durable energy.^[^ ^1^, ^2^, ^3^, ^4^, ^5^, ^6^, ^7^, ^8^ ^]^ As the successful commercialization of flexible/wearable electronics devices, extensive efforts have recently been devoted to develop various types of flexible power sources such as batteries^[^ ^9^, ^10^, ^11^ ^]^ and supercapacitors.^[^ ^12^ ^]^ Compared with traditional rigid energy storage devices, flexible lithium‐ion batteries (LIBs) have required to foldable, bendable, stretchable, and implantable, and therefore great challenges are remained in achieving both high energy density and deformation feature.^[^ ^13^, ^14^ ^]^

To improve flexibility and mechanical properties of flexible LIBs, various efforts have been made to design flexible materials and battery structures in the past several years.^[^ ^13^, ^14^, ^15^, ^16^ ^]^ First, a variety of flexible materials were developed in the electrodes and electrolytes, including carbon cloth, 2D materials, carbon nanofibers and 3D interlock structures coupled with gel electrolytes.^[^ ^17^, ^18^, ^19^, ^20^, ^21^, ^22^ ^]^ However, the improved flexibility of electrode materials is still limited to realize deformable and flexible batteries due to the stress concentration on the electrode materials and electrolyte, which greatly impact the ion transport behaviors. Alternatively, various novel configurations have been employed, such as wave‐like,^[^ ^23^ ^]^ ultrathin,^[^ ^5^ ^]^ cable,^[^ ^24^ ^]^ micro,^[^ ^25^ ^]^ textile^[^ ^26^ ^]^ and origami.^[^ ^27^ ^]^ These novel structural designs of flexible batteries could meet deformation features, while energy density (≈200 Wh L^−1^) was still limited for the practical application. At present, two types of single‐deformation flexible batteries based on structural design, i.e., spine‐like^[^ ^28^ ^]^ and zigzag,^[^ ^29^ ^]^ exhibited better energy densities of 242 and 275 Wh L^−1^, respectively. The comparison between flexible material design and flexible structure design suggests the latter would be more preferable to maintain the stable electrode kinetics under mechanical deformation.

To meet the practical requirement, scalable planar battery with both high energy density and favorable flexibility is still highly pursued. For achieving such goal, here a novel bidirectional snake‐origami battery of scalable planar structure was designed. With inspiration of rigid‐soft coupling features from chemical molecular structures, two types of functional units, i.e., rigid segment as energy unit and soft segment as deformation unit, were assembled into a flexible snake‐origami configuration. With robust electrode kinetics, the as‐fabricated battery has presented high energy density of 357 Wh L^−1^ (133 Wh kg^−1^), high‐rate capabilities and long‐term cycle, along with stable energy storage performance even under different mechanical deformation and continuous dynamic loads. Simulation results verified the validity of utilizing rigid‐soft coupled structure for enduring various deformations, and relationship between battery structure, energy density and flexibility could be established. The snake‐origami batteries with planar configuration exhibited multidirectional deformations and high energy density, which highlighted new perspective for achieving practical robust flexible batteries for wearable devices.

## 2 Results and Discussion

**Figure 1:**
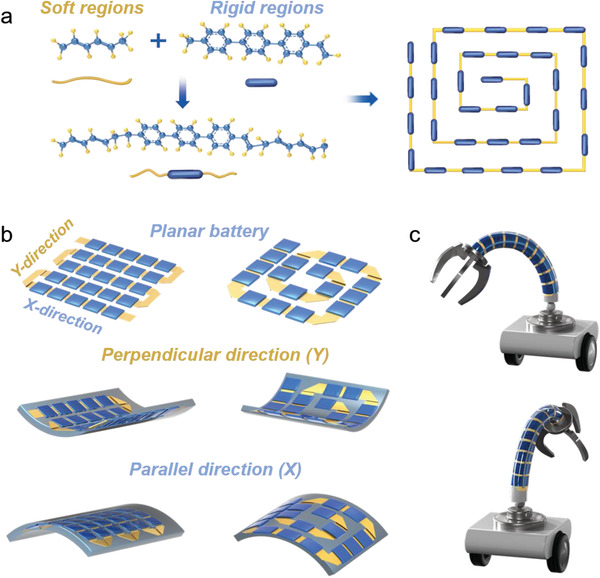
The schematic illustration of designing flexible snake‐origami batteries. a) Schematic illustration of the chemical molecular structures with soft and rigid regions; b) different types of snake‐origami batteries with bidirectional deformation; c) the snake‐origami batteries applied to bendable robot arm.

The organic chemical molecular structures (**Figure** **1**a) are composed of soft molecular chains and rigid molecular chains. The rigid molecular chains with high glass transition temperature, are played as a physical cross‐linking point and contributes to strength and hardness. The soft molecular chains have a low glass transition temperature, which contributes to a certain degree of flexibility and elasticity. Therefore, the rigid‐soft molecular chains can be designed as thermoplastic elastomer (TPE). In the design of snake‐origami batteries, the mechanical features of the organic chemical molecular structures (Figure 1a) are inspired to establish rigid and soft segments, which are responsible for energy storage units and flexible parts. Such a snake‐origami configuration would separate the energy storage and mechanical deformation segments, which allow the planar battery for endowing with both high energy density from the rigid segment and arbitrary deformation along with two directions (Figure 1b). To fulfill the high energy density, the novel electrode was fabricated into a come‐like shape used by kirigami design, as shown in Figure S1a (Supporting Information). Then, each comb tooth and separators were wrapped along with comb spine to form thick layered cells (Figure S1a, Supporting Information) and folded into bidirectional shape battery in Figure S1c (Supporting Information), and the rigid segments inspired from the molecular structure were constructed, which provided power sources over 90% (Figure S1, Supporting Information). The soft segments consist of gaps between comb teeth, and they occupied less than 10% area of the entire electrode/battery. The gaps were divided into two categories, i.e., wide and narrow gaps, which play the critical role in both connecting adjacent rigid segments and enabling mechanical deformation. Compared with the flexible battery module that several stiff lithium‐ion batteries linked in series by flexible wires, the snake‐origami batteries can avoid considering consistency of single battery. Wide gap (described as *a*) was applied to a U‐shaped fold or a perpendicular fold at the two ends of each lane, and thus the entire battery structure can be deformed along two directions, as shown in Figure S1b (Supporting Information). In a typical demonstration, the as‐designed bidirectional flexible snake‐origami batteries could be used to provide energy for robotic arms, enabling to grab stuff as illustrated in Figure 1c. Due to the planar configuration with bidirectional deformation ability, the snake‐origami batteries with high energy density could promote the design and manufacturing level for achieving practical flexible batteries in wearable devices.

**Figure 2:**
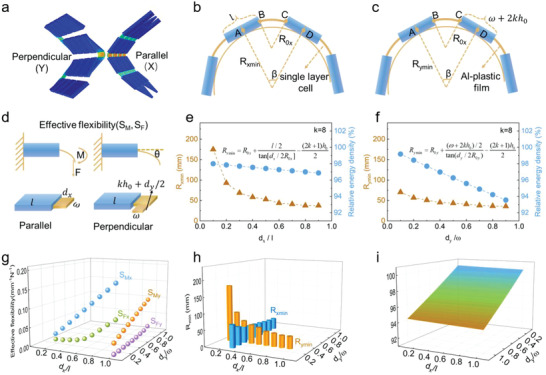
The theory and simulation analysis of snake‐origami batteries. a) Finite element calculations of snake‐origami batteries under bending state with bidirectional deformation: parallel (*X*) and perpendicular (*Y*) directions; Schematic illustration of the minimum bending radius along the b) parallel direction and c) perpendicular direction. d) The effective flexibility of snake‐origami batteries with bidirectional. The battery was considered as composite laminated cantilever beam. e,f) The relationship between the energy density and the minimum bending radius as a function of dimensionless gap width *d_x_*/*l* or *d_y_*/*ω* upon bidirectional bending. g) The effective flexibility, h) minimum bending radius, and i) relative energy density varies with structure parameters of the snake‐origami batteries.

For fundamentally understanding the advantage of such snake‐origami batteries, the design principles toward balancing flexibility and electrochemical performance under mechanical deformation were discussed. To clarify mechanical durability of the snake‐origami batteries, finite element calculations were implemented to analyze the deformation at soft connecting segments. The results in **Figure** **2**a show that the snake‐origami battery with a 4 × 4 array can bend along with two directions. The stress was concentrated on the soft segments under bending deformation. In the battery structures, the battery element was treated as the composite laminated cantilever beam. Then, bending radius (Figure 2b,c) was used to describe mechanical durability of the battery in both the two directions.^[^ ^30^ ^]^ The batteries were wound around the semicircle by bending it, equivalent to its actual bending state in practical applied scene. Under the bending state, the bending radius was corresponded to the critical state of plastic deformation of the battery. It should be noted that the flexible snake‐origami batteries cannot be folded 180 degrees, which will cause irreversible damage to the battery. With the decrease of bending radius, there must be a minimum bending radius (*R*_min_) which the soft segments could exactly reach in the bending procedure. To further ensure the yielding location in the battery, the minimum bending radius calculations under single bending moment were carried out, as shown in Figure 2b,c. The changes of *R*_min_ in both directions were smaller even though the winding layer number (*k*) increased (Figure S5, Supporting Information). The minimum bending radius was decreased with the gap increasing (Figure 2e,f). The results show that the flexibility and energy density are in the competitive status. The bent ability was evaluated by applying concentrated force or a bending moment, and the element was selected in each direction (Figure 2d; Figure S3, Supporting Information). Effective flexibility was defined to evaluate the flexibility of battery, and *S*_M_ and *S*_F_ were defined as the effective flexibility at different directional bending deformation and under concentrated force, respectively.^[^ ^30^ ^]^ According to Figure 2g, effective flexibility in both directions shows similar behaviors, which suggests that *S*_M_ and *S*_F_ increased as dimensionless gap width *d_x_*/*l* or *d_y_*/*ω* increased under bending deformation. However, the relationship between effective flexibility and winding layer number exhibited different laws in the *X*‐ and *Y*‐directions, as shown in Figures S6 and S7 (Supporting Information). The effective flexibility was not varied with increasing winding layer number in the *X*‐direction. Yet the effective flexibility would be improved when the winding layer number increases in the *Y*‐direction. The relative energy density (*E*_R_) is a parameter that is used to compare energy density of the flexible battery with conventional battery. Similarly, the relative energy density (*E*_R_) was closely related to the structure parameters as shown in Figure S4 (Supporting Information). And the energy density of flexible battery is lower than that of conventional battery. When any structure parameters changed, the relative energy density also changed (Figure S4, Supporting Information). The *d_x_*/*l* and *d_y_*/*ω* exhibited the greatest impact on *E*_R_. The smaller minimum bending radius would result in improved effective flexibility. On the contrary, the improved flexibility of the battery leads to lower relative energy density. All these features depend on the structure parameters, as shown in Figure 2g–i. Therefore, design of snake‐origami batteries was dependent on structure parameters, and the key parameters energy density, bending radius and effective flexibility should be considerably concerned, which can be concluded form Figure 2 and Figures S1–S7 (Supporting Information) in theory. The best flexible battery should be having excellent effective flexibility, lower bending radius and highest energy density. According to the above relations of meeting various flexible devices, systematic design is still a tremendous trend in flexible batteries.

**Figure 3:**
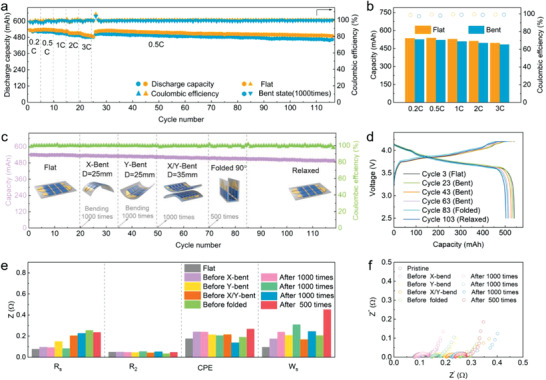
The electrochemical performance of snake‐origami batteries with various deformations. a) Rate and long cycle performance (1 C = 540 mA). b) Average capacity at different current densities. The cycle performance c) and charge–discharge curves d) of snake‐origami batteries with different states: flat, bent, fold, and relaxed state under constant current density of 0.5 C. e) The typical resistance of each component for various state according to the equivalent circuit model. f) Nyquist plots of the EIS spectra.

In this work, considering the operability of the assembly process and performance of battery, snake‐origami batteries with 3 × 3 array were assembled, which was used to verify the feasibility of novel flexible battery design. To demonstrate robust electrochemical performance under mechanical deformation, the snake‐origami batteries were assembled with LiCoO_2_ (as positive electrode material) and graphite (as negative electrode material), followed by testing under flat and bent states at different current densities. As exhibited in **Figure** **3**a, the snake‐origami batteries with both bent and flat states showed very similar excellent rate performance and long‐cycle stability. In first half of Figure 3a, the snake‐origami batteries were tested under different current density to exhibit rate performance. Then we used this same snake‐origami batteries to test cycle performance under current density of 0.5 C. Considering timeliness and polarization, current density of long cycle performance was settled as 0.5 C. Under bent state at different current densities, the average discharge capacity was also similar with that of the values achieved at flat state (Figure 3b), and the capacity could be well maintained with no pronounced capacity drop under bent state (over 92.5%) (Figure S8, Supporting Information). After bending 1000 times with a bending diameter of 25 mm, the capacity of bent snake‐origami batteries presented limited capacity decrease in comparison with initial flat state. Such good capacity stability at high current density should be attributed to the bidirectional design, allowing the battery structure for remaining tight contact between each layer. The slightly decreased capacity can be attributed to the change of the soft segments (also with gaps for storing certain electrolytes), which play as the roles of joint and mechanical deformation. Thus, the ion transport in the electrolyte near the rigid regions may be changed in the snake‐origami batteries of bending for many times. To clarify the excellent electrochemical performance of snake‐origami batteries, electrochemical impedance spectra (EIS) was additionally employed to test under different states (Figure S9a,b, Supporting Information). The EIS presented a slight shift under bent state, which may be caused by the variation in the ion transport process. Subsequently, an equivalent circuit was applied to fit EIS to describe impedance changes of each component (Figure S9e and Table S1, Supporting Information). According to equivalent circuit model, *R*_s_ is mainly related with ohmic resistance of electrolyte and internal resistance of the electrodes. *R*_2_ and CPE are dependent on the resistance of charge transport and corresponding capacitance at the electrode/electrolyte interfaces, respectively. Warburg resistance (*W*_s_) relies on the ion diffusion in the solid electrodes. Note that *R*_s_ became smaller upon bent due to the tight interface contact. However, the value of *W*_s_ and CPE became larger, which implies slightly increased ion diffusion and increased capacitive nature of the battery. Furthermore, the distribution of relaxation time (DRT) method was further implemented to verify the EIS results (Figure S9c,d, Supporting Information). The behavior in each frequency range exhibited identical tendency with resistance of equivalent circuit model, and the battery under flat and bent status presented stable kinetics process. Therefore, the increased resistance leads to a capacity decrease in the snake‐origami batteries under bent state, which is attributed to the varied ion transport behaviors.

For evaluating the performance under practical applications, the snake‐origami batteries were used to operate under various mechanical deformations (Figure 3c; Figure S11, Supporting Information), such as bent, folded and flat states. First, the snake‐origami batteries were charged and discharged for 19 cycles in the flat state, and the discharge capacity slightly decreased ≈1.3% (0.086% every cycle) from 540 to 533 mAh (Figure 3c). Then, the snake‐origami batteries were bent for 1000 times with a diameter (*D*) of 25 mm in the *X*‐direction, while it operated for 15 cycles along with a capacity ≈530 mAh close to the value achieved in the flat state. Because the snake‐origami batteries can perform deformation along both directions, the as‐fold batteries were bent in the *Y*‐direction, showing a retained discharge capacity of ≈523 mAh (98.7% of 530 mAh). This indicates that the mechanical deformation almost has no meaningful impact on the energy storage performance of the snake‐origami batteries. Additionally, the snake‐origami batteries were bent for 1000 times with *D* = 35 mm in both directions, while they were charging and discharging for 20 cycles, and then the snake‐origami batteries were folded 500 times under 90° angle, followed by cycling 15 cycles. With undergoing a series of mechanical deformations, the discharge capacity of snake‐origami batteries presented good capacity retainment. The results suggest that the average capacity loss was estimated to be 0.066% for each cycle under different mechanical bending conditions. At last, the batteries continued for stable cycle with the average capacity of 500 mAh. The excellent cycle performance can be attributed to the introduction of soft segments, which were used to deform under mechanical stress. While the snake‐origami batteries cannot be twisted, due to the structural design of the battery. When the battery is twisted, the rigid segments (energy storage units) were damaged that affect electrochemical performance, as shown in red regions of Figure S13 (Supporting Information).

Additionally, the discharge–charge curves in Figure 3d presented the capacity and voltage change, showing very small overpotential change. In order to understand the electrochemical kinetics, EIS was implemented to snake‐origami batteries under different deformations (Figure 3f), and the EIS showed very limited change in the impedance. The curves in Figure 3f shows that the battery exhibited higher real axis values at the high frequency intercept as the number of cycles increased that was consistent with results in the typical resistance of the equivalent circuit model. The real axis intercept is often associated with the solution resistance, interface resistance *R*_s_. The *R*_s_ decreased with cycling has resulted in the ratio of capacity decrease. Equivalent circuit model and DRT method were carried out to further analyze EIS results, as shown in Figure 3e and Figure S12 (Supporting Information). The values of CPE and *W*_s_ became larger and indicates slightly increased impedance in the ion diffusion process, which corresponded to DRT analysis results. The snake‐origami batteries with different mechanical deformations may cause change in the local electrolyte distribution and ion transport, which is linked with resistance variation.

**Figure 4:**
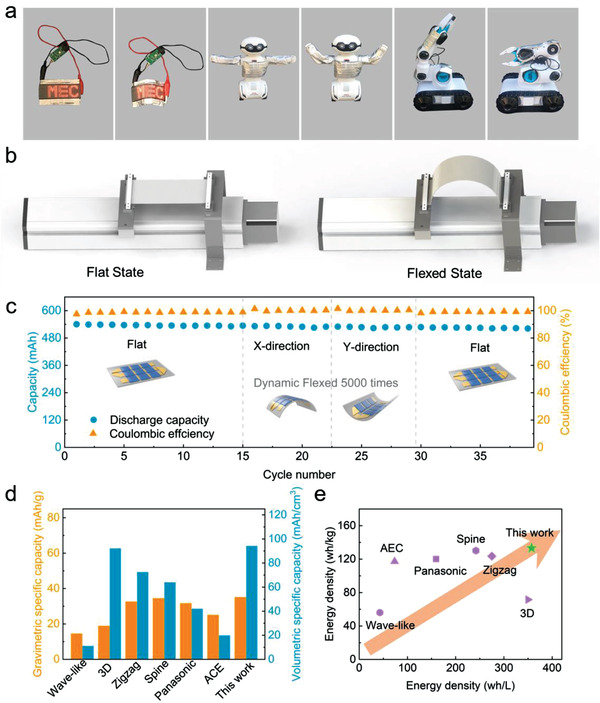
a) The snake‐origami batteries were applied to power a flexible LED display screen, a robot, and a mechanical arm. b) Schematic illustration of dynamic loading devices, and the electrochemical performance was tested under dynamic loading. c) Cycle performance of the snake‐origami batteries under continuous mechanical loading at 0.5 C. *X*‐axis was described as cycle numbers of battery working. d) The state‐of‐the‐art of cell‐scale energy density for the reported flexible batteries, along with comparison of energy density in snake‐origami batteries (ACE: Apower Electronics Co., Ltd). e) Cell‐scale specific capacity of previous reported flexible batteries compared with snake‐origami batteries.

Furthermore, the snake‐origami batteries were used to provide energy for LED display screen and smart devices to demonstrate practical application. The flexible LED screen with a pattern “MEC” was lighted with the battery under different deformations (**Figure** **4**a; Figure S14, Supporting Information). The videos (Videos S1–S4 in the Supporting Information) were recorded to illustrate that snake‐origami batteries could be deformed with flexible LED screen, and the deformable snake‐origami batteries can still power the LED screen. While Videos S1 and S2 (Supporting Information) showed LED display screen powdering by snake‐origami batteries under dynamic loading in the *X*‐ and *Y*‐directions, respectively. Similarly, Videos S3 and S4 (Supporting Information) exhibited flexible devices that flexible screen together with snake‐origami batteries under dynamic loading in the *X*‐ and *Y*‐directions, which were closed to the actual working environment. The snake‐origami batteries could be applied to the robot and robot arm with an appropriate circuit design, which can adapt the deformation with working smart devices.

In the case of the snake‐origami batteries applied to practical environment, such as flexible wearable device, medical and artificial skin, they may undergo continuous mechanical deformations in the entire life cycle. Therefore, the mechanical endurance of snake‐origami batteries with continuously loads would be a critical factor to evaluate flexibility and electrochemical performance stability. The cycle performance with continuous deformation of the snake‐origami batteries should be tested under dynamic bending load with a certain moving distance, upon simultaneous charging/discharge at constant current density. The scheme of the dynamic load device was illustrated in Figure 4b. In Figure 4c, the snake‐origami batteries were first charged and discharged at 0.5 C for 15 cycles with flat state. After operating for 15 cycles, the discharge capacity was about 535 mAh with Coulombic efficiency of 99%. Then, dynamic mechanical load was applied to battery in both directions with a speed of 1.6 mm s^−1^, and the moving distance was 50 and 35 mm along the *X*‐direction and the *Y*‐direction, respectively. The total bending times were about 5000 times. Due to dynamic flexed, the discharge capacity was slightly decreased to 530 mAh, suggesting that the dynamic flexed has almost no impact on the capacity. The details of the discharge–charge curves were obtained under flat and dynamic flex state from Figure S15 (Supporting Information). Compared with the results obtained from flat state, the discharge–charge curves were slightly fluctuated and the overpotential was varied with the dynamic flex at different states, while Coulombic efficiency could be remained around 99%. Voltage fluctuation can be attributed to the change of the electrolyte distribution at certain local regions, and ions transport varies with dynamic flex. This phenomenon indicates that dynamic flex has limited effect on the electrochemical performance, due to tight contact between the electrodes and current collectors. After dynamic loads, the snake‐origami batteries under flat state could remain stable cycle performance. In the further efforts, the snake‐origami batteries should be specifically designed to accommodate the dynamic loads in the soft segments. In order to evaluate the changes occurring due to bending the battery, the electrochemical performance of battery with or without deformation was analyzed. Form Figure S10 (Supporting Information), it stated that the deformations have almost no effect on cycle performance duo to the similar specific capacity changes. It is unclear that cycle working has a predominant effect in the electrochemical performance.

Noticeably, the volume energy density of the snake‐origami LIB was 357 Wh L^−1^ with a volume of 5.66 cm^3^ and the specific energy density of 133 Wh kg^−1^ with weight of 15.2 g. Compared to the previous reported on cell‐scale flexible batteries from both academic and industry (Table S3, Supporting Information), the snake‐origami batteries here exhibited high energy density, which is compatible with the commercial requirement in the flexible devices. While in order to make a diversified comparison and better show the advantages of snake‐origami batteries, most kinds of flexible batteries were chosen in different perspectives, such as single layer battery and multilayers battery. The results in Figure 4d,e suggest that the snake‐origami batteries in the present study hold much improved capacity and energy density, highlighting a record‐setting values achieved in the cell‐scale flexible LIBs.

It is noted that there are great advantages using the snake‐origami prototype for designing a novel type of novel planar batteries. Initially, inspiration of the decoupled rigid‐soft molecular structures provides a new perspective of designing planar flexible batteries with both high flexibility and energy density. Therefore, the separation of energy storage units and mechanical deformation parts would provide a unique feature in battery design. The energy storage units could be specifically designed and integrated, aiming at achieving superior energy density, and meanwhile the mechanical deformation parts could be also manipulated to meet unique deformation behaviors in the practical cases. More importantly, the snake‐origami configuration would be significant design in achieving deformation along different directions, aiming to increasing degree of freedom while the planar battery feature could be well retained. For well guiding such design, on the other hand, a design principle was also proposed. Typically, key parameters including geometric factors, relative energy density, bending radius and effective flexibility are concerned, with purpose of generating the balance between energy density and deformation ability. Such principles would be not only valid in designing flexible snake‐origami batteries in the present study, while could be also extended to design other configurations of flexible batteries. Furthermore, owing to the novel design and rational fabricating using kirigami–origami techniques, the constructed flexible snake‐origami LIBs could well present the advancement in both energy storage capability (record‐setting value achieved) and flexibility along with two directions. While in the novel design of the snake‐origami batteries cannot be bent at the same time in two directions that would cause irreversible deformation of the battery, and can only bend in single directions at the same time. The manufacturing processes mainly involve the cutting and folding operation procedures, which could be scaled up for large‐scale manufacturing with a specifically designed fabrication machine. Furthermore, the flexibility of battery could be further improved by minimizing the size of each region in this design of snake‐origami configuration. Overall speaking, combination of the snake‐origami design, design principle and fabrication techniques presented in this study highlights the opportunities for achieving novel high‐performance flexible planar LIBs.

## 3 Conclusion

In summary, a novel prototype of planar flexible snake‐origami LIBs was demonstrated with inspiration of molecular structures. The decoupled design of the rigid and soft regions was employed to construct energy storage segments and mechanical deformation parts, respectively. For rationally evaluating the LIBs, design principles were proposed using finite element calculations, suggesting the intrinsic link between the energy density and geometric structure parameters. The as‐fabricated snake‐origami LIBs exhibited superior energy density of 357 Wh L^−1^ (133 Wh kg^−1^), along with robust energy storage performance even under different mechanical deformation and continuous dynamic bending for 5000 times. Apparently, both the design concept and LIB fabrication here constructed a platform for boosting the development of high‐performance scale‐cell flexible LIBs for practical applications.

## Conflict of Interest

The authors declare no conflict of interest.

## Supporting information

Supporting Information

Supplemental Video 1

Supplemental Video 2

Supplemental Video 3

Supplemental Video 4

## Data Availability

Research data are not shared.
