# Supplementary material for: Bidirectional Planar Flexible Snake‐Origami Batteries
Source: Adv Sci (Weinh). 2021 Aug 27;8(20):2101372. doi: 10.1002/advs.202101372 (PMC8529459; doi:10.1002/advs.202101372)

© 2021 Wiley-VCH GmbH

ADVANCED SCIENCE
Open Access

Supporting Information

for *Adv. Sci.,* DOI: 10.1002/advs.202101372

Bidirectional Planar Flexible Snake-Origami Batteries

Na Li, Haosen Chen, Shuangquan Yang, Heng Yang, Shuqiang Jiao, and Wei-Li Song**

Supporting Information

Bi-directional planar flexible snake-origami batteries

Na Li, Haosen Chen[*], *Shuangquan Yang, Heng Yang, Showing Jiao*, *Wei-Li Song*[*]

N. Li, Prof. H. Chen, Prof. W.-L. Song, S. Yang, H. Yang, Prof. S. Jiao

Institute of Advanced Structure Technology, Beijing Institute of Technology, Beijing 100081, China
E-mail: weilis@bit.edu.cn; chenhs@bit.edu.cn; sjiao@ustb.edu.cn;

N. Li, Prof. H. Chen, S. Yang, H. Yang, Prof. W.-L. Song

Beijing Key Laboratory of Lightweight Multi-functional Composite Materials and Structures, Beijing Institute of Technology, Beijing 100081, P. R. China

Prof. S. Jiao,

State Key Laboratory of Advanced Metallurgy, University of Science and Technology Beijing, Beijing 100083, P. R. China.

1. Experimental section

1.1 Battery assembly

Commercial $LiCoO_2$ electrodes and graphite electrodes were provided by Jiangsu HaiTao new energy technology Company, used as positive electrode and negative electrode, respecitively. First, all electrodes and ceglard separators were cut into comb-like shape with designed geometric size, as shown in Figure S1(a). Then the electrodes and separators were wound with comb spine in Figure S1(a) and folded into bi-directional shape battery in Figure S1(c). The batteries were dried in a vacuum oven at 80 °C for 12h. After drying, the batteries were injected electrolyte in Ar_2 filled glovebox (O_2 <0.1 ppm, H_2O<0.1ppm). The electrolyte was 1mol/L $LiPF_6$ in the EC (ethylene carbonate)/DMC(dimethyl carbonate) with volume ratio of 1:1(*DoDoChem*). After 4h infiltration, the batteries were sealed by aluminized plastic film under vaccum environment.

In this work, considering the operability of the assembly process and performance of battery, snake-origami batteries with 3*3 array were assembled and tested. In the Figure S4, When any structure parameters changed the relative energy density also changed and the d_x/l and d_y/ω exhibited the greatest impact on E_R, thence3 3*3 array was choosed. And we set the $d_x/l= d_y/\omega$=0.2. This design of snake-origami batteries have better relative energy density and flexibility form the Figure 2, when $d_x/l= d_y/\omega$=0.2.

The actual geometric size of above assemblied battery was shown in Figure S2. The snake-origami batteries with 3*3 array were assembled in Ar_2 filled glovebox. All the structure parameters of snake-origami batteries were idential that $Nx=Ny$=3 and the winding layer numbers was about 6-7. The width of the gap in X- and Y-direction

are both 2 mm. ω was the width of comb spine in the battery, which is 10mm. The width of the comb tooth is 10 mm， which l=10 mm. Bending radius and shift can describe the bending state more reasonably. Different bending tests were run sequentially on the snake-origami batteries, which were closed to practical working envrionment of flexible battery. The snake-origami batteries were cycled 15 cycle, about four days, after every bending test. The test steps were as follows. The batteries rest 2h after cycling, then bending test. After bending test, the battery cycled after resting 2h.

1.2 Electromchemical test

The snake-origami batteries used $LiCoO_2$ and graphite electrode were tested under the voltage between 2.5V and 4.2V by charge/discharge battery testing of Neware instruments. The assembled batteries were tested indoor to maintain relatively constant temperature and humidity. The assembled snake-origami batteries were measured at room temperature, about 25°C. And the relative humidity of the air in the room is about 30%. The batteries were chareged to 4.2V at constant current and held at 4.2V until current was reduced to 0.025C. Then, the batteries were discharged to 2.5V. The electrochemical impedance spectroscopy (EIS) measurements were tested by a BioLogic VMP3 instruments. The requency range of EIS was from 10^6 Hz to 10^{-1} Hz.

While commerical lithium-ion batteries are mainly measured by battery capacity (mAh) and energy density (Wh). This flexible battery device used commerical $LiCoO_2$ electrodes and graphite electrodes, which were provided by Jiangsu HaiTao new energy technology Company. The uniformity of commerical electrodes is relatively good, which have same areal density and specific capacity. This flexible battery chose

capacity as the evaluation criterion to show the consistency of battery capacity. Specific capacity can be obtained by calculating the quotient of battery capacity (mAh) and the quality of the electrodes.

1.3 Numercial simulation

The mechanical deformation of snake-origami batteries was analyzed by three-dimensional standard nonlinear finite element method in the commercial software ABAQUS. In all cases, four-node quadrilateral stress/displacement elements with reduced integration were used. For simplicity, linear isotropic elasticity was adopted for the battery structure with effective modulus and Poisson ratio based on experimental parameters. The pressure of 1 atm was applied to both sides of deformable layers to simulate the vacuum conditions inside the aluminized plastic film. The simply supported boundaries were adopted at the ends of structures and the cylinder was fixed.

2. Theory energy density calculated

2.1 Calculated the theoretical energy density of snake-origami batteries

The area specific capacity of $LiCoO_2$ positive electrode was 2.90mAh/cm^2 and mass loading of it was 21.2mg/cm^2(double coated). The thickness of positive was 80μm. The area specific capacity and mass loading of graphite negative electrode were 3.40mAh/cm^2 and 10.52mg/cm^2(double coated), respectively. The thickness of negative was 86μm.The ratio of negative/positive was 1.17 and the negative was excessive. The weight of snake-origami batteries was 15.2g, including electrode, electrolyte and aluminized plastic film.

From the Figure S1, the geometric size of the snake-origami batteries was shown.

The width L: $L = 2 \times 2 + 1 \times 7 + 0.2 \times 3 \times 2 + 3.2 \times 2 = 18.6cm$

The length L_{R}: $L_R = 16cm$

The thickness of separator was 15µm and the thickness of aluminized plastic film (single layer) was 113µm.

Therefore, total area of $LiCoO_2$ positive was183.6cm^2.

$$S_{Positive} = 16 \times (2 \times 2 + 1 \times 7) + 1 \times (0.2 \times 3 \times 2 + 3.2 \times 2) = 183.6cm^2$$

So, the capacity of battery was 532.4mAh.

$$2.9 \times 183.6 = 532.4mAh$$

The width of the sealed edge is 2 mm and 20mm. The thickness of the edge part is 0.25 mm. After assembled, the length of snake-origami batteries without extra edge is 55mm and with extra edge is 75mm. The width of snake-origami batteries without extra edge is 44.5mm and with extra edge is 46.5mm.

The total surface area of snake-origami batteries was 34.87cm^2.

$$S = 46.5 \times 75 = 3487.5mm^2 = 34.87cm^2$$

The total volume of snake-origami batteries was 5.66cm^3.

$$V = 11 \times 10 \times 13.5 \times 3.33 + 13 \times 13 \times 2 \times 0.9 + 55 \times 0.2 \times 0.25 \times 2 + 6 \times 10 \times 2 \times 0.45 + 75 \times 2 \times 0.25 + 20 \times 46.5 \times 0.25 + 13 \times 13 \times 2 \times 0.25 = 5663.25mm^3 = 5.66cm^3$$

The theory energy density of snake-origami batteries was calculated as follows. 3.8V is the average discharge voltage of the snake-origami batteries, which is similar to the nominal voltage of $LiCoO_2$. The average discharge voltage was calculated by the ratio of energy and capacity, which can be obtained from the charge-discharge of Figure 3d.

Area Capacity Density: $E_S = 532.4 \div 34.87 = 15.27mAh/cm^2$

Volume Energy Density: $E_V = 532.4 \div 5.66 \times 3.8 = 357.44 Wh/L$

Gravimetric Energy Density: $E_m = 532.4 \div 15.2 \times 3.8 = 133.1 Wh/kg$

2.2 Relative energy density

As shown in Figure S1(a-b), the width and length of electrode is L and $L_{R,}$ respectively. ωwas the width of comb spine in the battery. The gaps of different direction were defined as d_x (X-direction) and d_y (Y-direction). In the Figure S1b, the wide gap was described as a, which was used to folded insipred by origami in the Y-direction of snake-origami batteries. For the snake-origami battery, N_x and N_y were the number of rigid segment in X- and Y-direction. The E represents the energy density of the snake-origami batteries in Figure S1(a). The E_{total} is descirbed as the energy density of conventional battery assembled by the complete electrode in Figure S1(b). As shown in Figure S1(c), k was the winding layer numbers of the snake-origami batteries and conventional batteries and e_0 was the area capacity of positive electrode. S and V were the total surface area and volume of the snake-origami batteries, respectvely. Relative energy density can be calculated by the ratio of E/ E_{total}.

The length of electrode in the battery L_R:

$$L_R = (2k+1)^2 h_0 + (2k+1)\omega$$

The wide gap of conventional battery:

$$a = 2\omega + d_y + 2kh_0$$

The thickness of single layer battery:

$$h_0 = h_{cathode} + h_{separator} + h_{anode}$$

The thickness of battery in Figure S1(a-b):

$$h = (2k+1)h_0$$

The energy density of the snake-origami batteries:

$$E = \frac{e_0 S}{V} = \frac{[(N_x.N_y+2)l.L_R+\omega(N_x-1)d_x N_y+(N_y-1)(2\omega+d_y+2kh_0)\omega]e_0}{(N_x N_y+2)l(\omega+2kh_0)(2k+1)h_0+(N_x-1)d_x\omega h_0 N_y+a\omega h_0(N_y-1)+(N_y-1)[N_x l+(N_x-1)d_x]d_y h_0}$$

The energy density of the conventional batteries:

$$E_{total} = \frac{e_0\{[(N_x N_y+2)l+(N_x-1)d_x N_y]L_R+(N_y-1)\omega a\}}{[(N_x.N_y+2)\ l+(N_x-1)d_x N_y](\omega+2kh_0)(2k+1)\ h_0+(N_y-1)\omega a h_0}$$

The relative energy density was defined as

$$E_R = \frac{E}{E_{total}} =$$

$$\frac{\{[(N_x N_y+2)l+(N_x-1)d_x N_y](2k+1)h_0(\omega+2kh_0)+a\omega h_0(N_y-1)\}}{\{(N_x N_y+2)l(\omega+2kh_0)(2k+1)h_0+(N_x-1)d_x\omega h_0 N_y+a\omega h_0(N_y-1)+(N_y-1)[N_x l+(N_x-1)d_x]d_y h_0\}}\frac{[(N_x.N_y+2)l.L_R+\omega(N_x-1)d_x N_y+(N_y-1)\omega a]}{\{[(N_x N_y+2)l+(N_x-1)d_x N_y]L_R+(N_y-1)\omega a\}}$$

Figure S1. Schematic illustrations of the structure parameters of electrode and flexible snake-origami battery. (a) The comb-like electorde (top) of snake-origami batteries and each comb tooth and separators were wrapped along with comb spine to form thick layered cells (bottom). (b) The complete electrode without undesigned and cut into many strips (top) and The battery (bottom) was assembled by the top electrode. (c) The snake-origami batteries and the structure parameters.

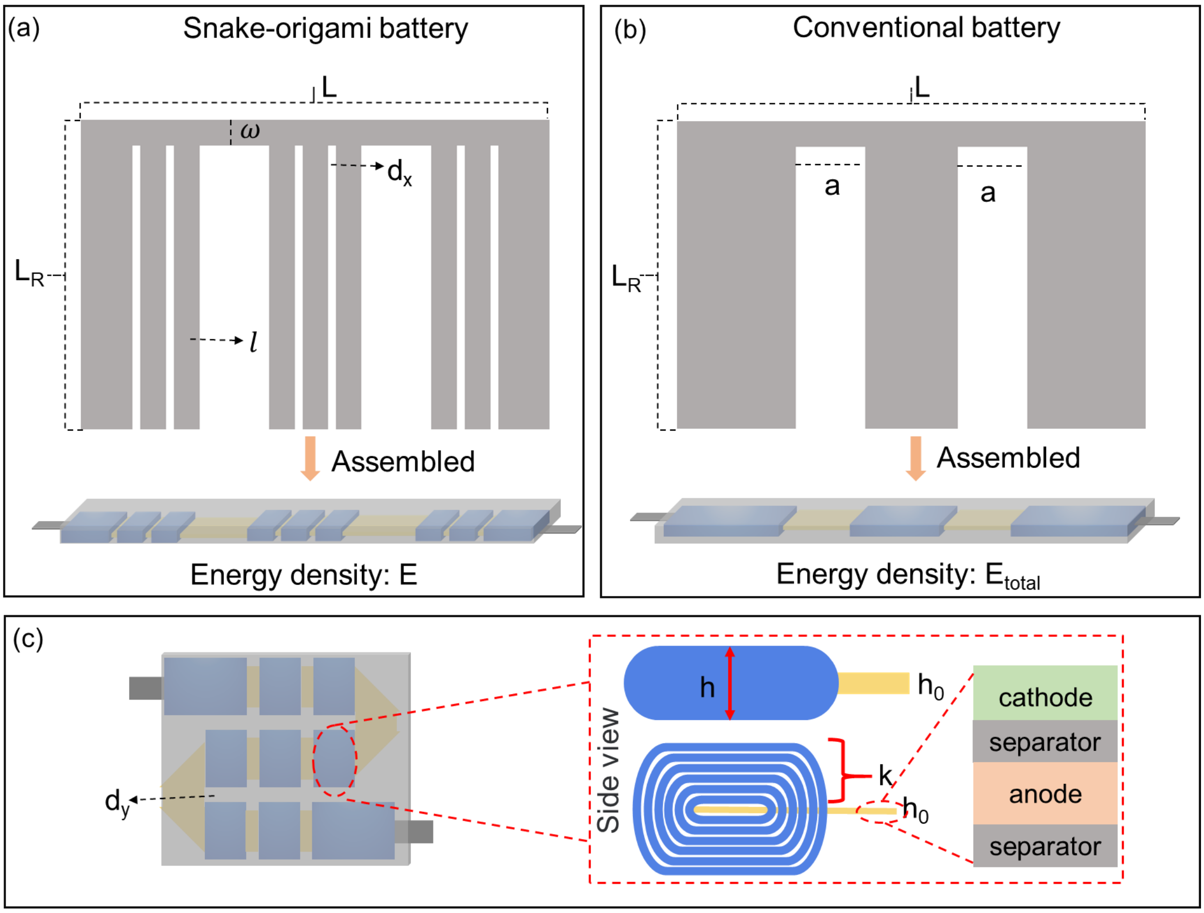

Figure S2. (a) Schematic illustration of the actual geometric size of snake-origami battery (Units: mm). (b) Image of real battery after assembled.

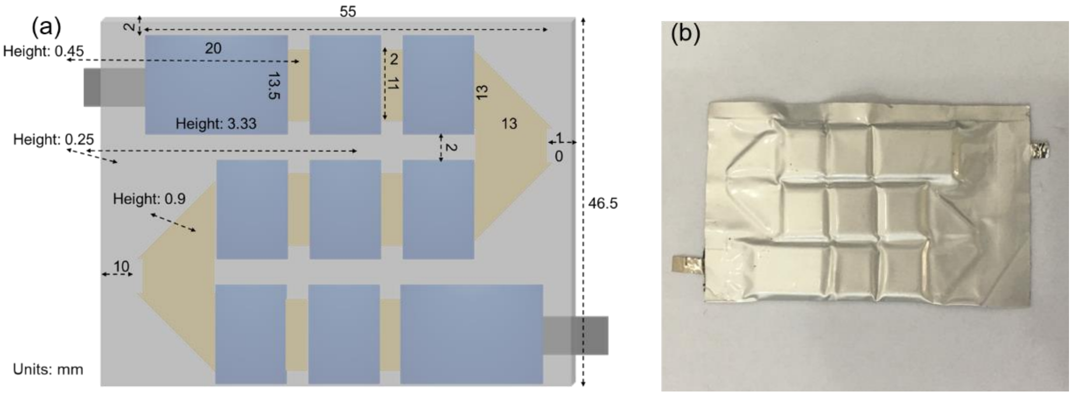

2.3 Effective Flexibility

In order to describe the flexibility of battery, effective flexibility was defined. The effective flexibility was a function of geometric structure parameters, under different mechanical deformation. The equation of effective flexibility was exhibited as followed.

Figure S3. Schematic illustrations of the effective flexibility of snake-origami batteries.

Parallel direction (X-direction)

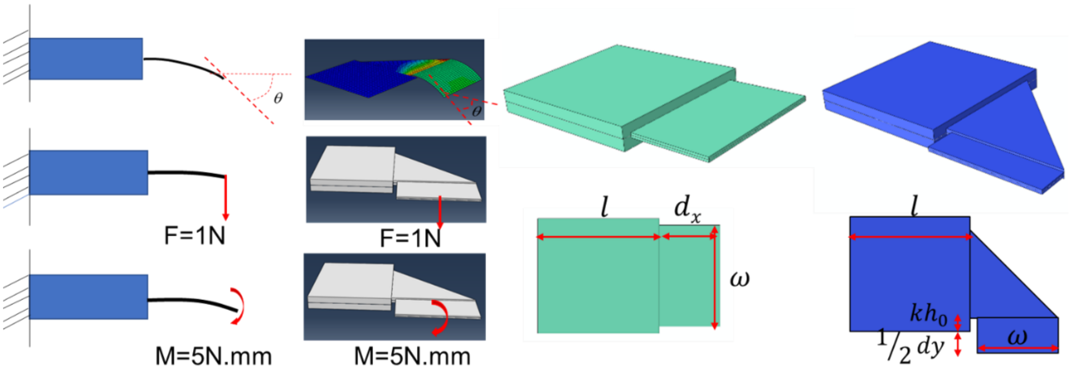

$$S_{Fx} = \frac{\omega\theta}{Fl^2}$$

$$S_{Mx} = \frac{\omega\theta}{Ml}$$

Perpendicular direction (Y-direction)

$$S_{Fy} = \frac{a\theta}{F\omega^2}$$

$$S_{My} = \frac{a\theta}{M\omega}$$

S_{Mx} and S_{My} were defined as the effective flexibility at different directional bending deformation.

S_{Fx} and S_{Fy} represented the effective flexibility under concentrated force.

Figure S4. The relationship between relative energy density and geometric structure parameters. The number of rigid segment in different direction: (a) N_x and (b) N_y. The relative energy density varied with dimensionless d_y/ω (c) and d_x/l (d), when $d_x=d_y=3mm$ and $N_x=N_y=5$. (e) The relative energy density was a function of k with different gaps and number of rigid segments.

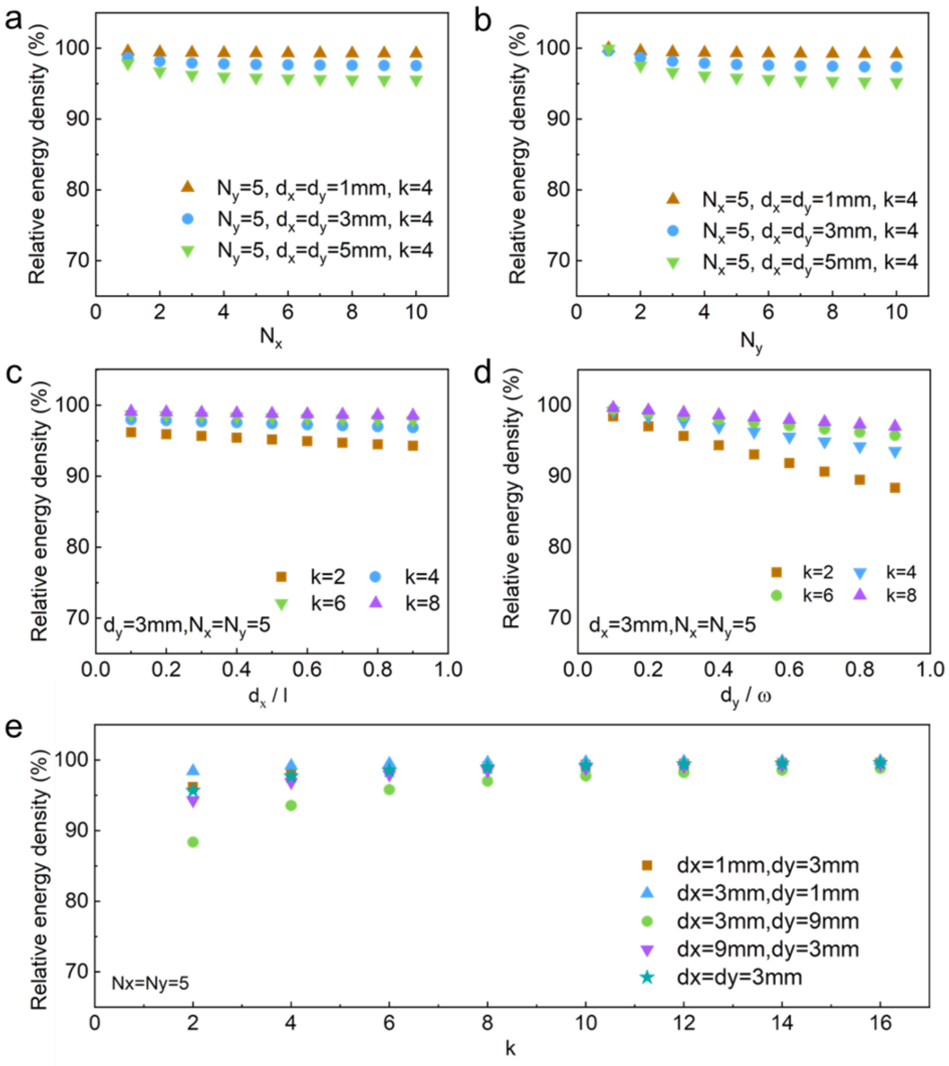

Figure S5. The relationship between minimum bending radius and geometric structure parameters: gap (d_x, d_y) and winding layer numbers (k). The minimum bending radius varied with dimensionless gap width d_x/l (a) and d_y/ω (b), under different winding layer numbers.

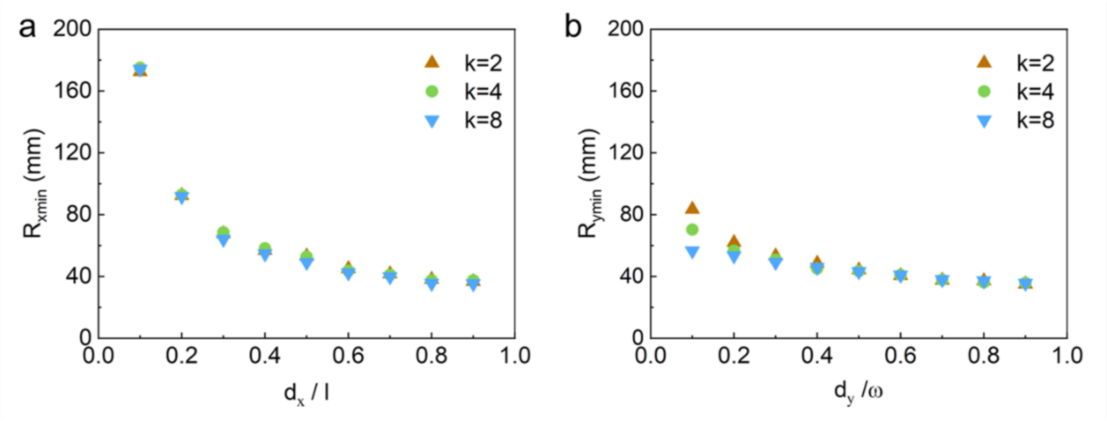

Figure S6. The relationship between effective flexibility and dimensionless gap width d_x/l or $d_y/\omega$with different direction: X-direction (a, b) and Y-direction (c, d).

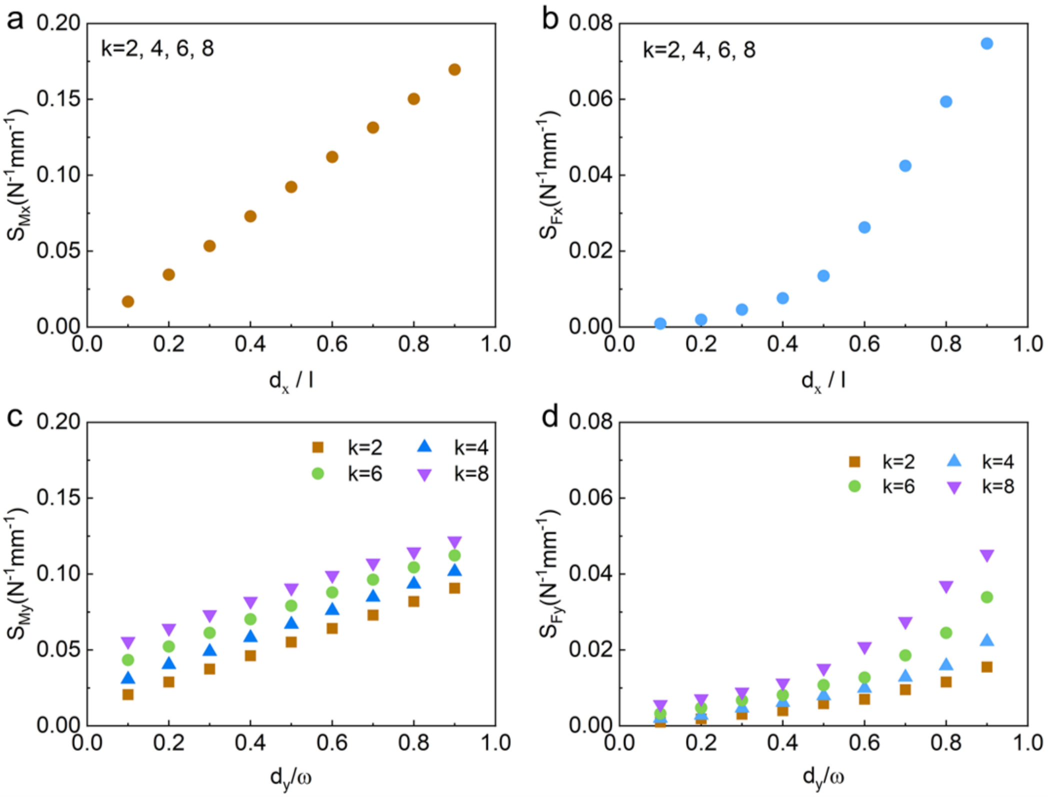

Figure S7. The effective flexibility was a function of winding layer number with dimensionless gap width: X-direction (a, b) and Y-direction (c, d).

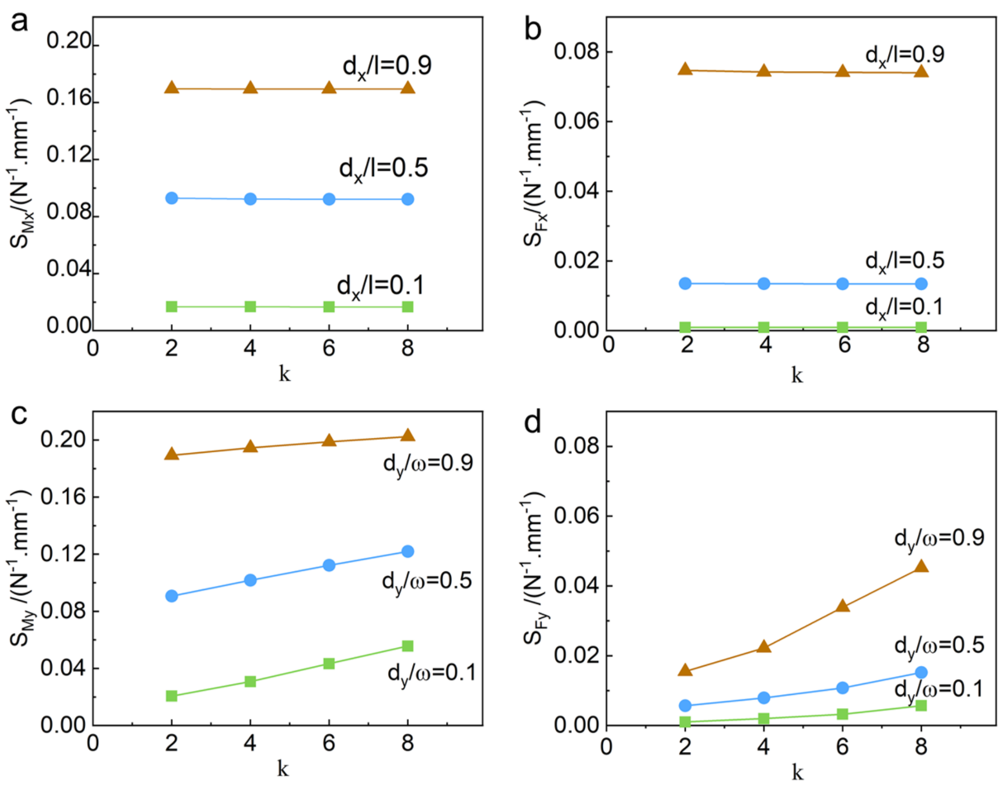

Figure S8. The capacity retention rate at different current densities.

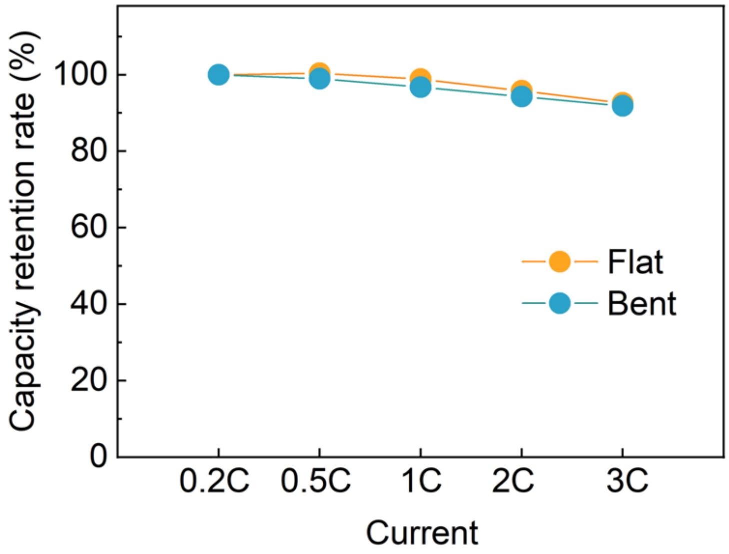

Figure S9. Nyquist plots of the EIS spectra with different states: (a) Pristine and (b) bend 1000 times (D=25mm). Distribution of relaxation time (DRT) results after treated the EIS spectra data: (c) Pristine and (d) bend 1000 times. (e) The typical resistance of each components for various state according to the equivalent circuit model.

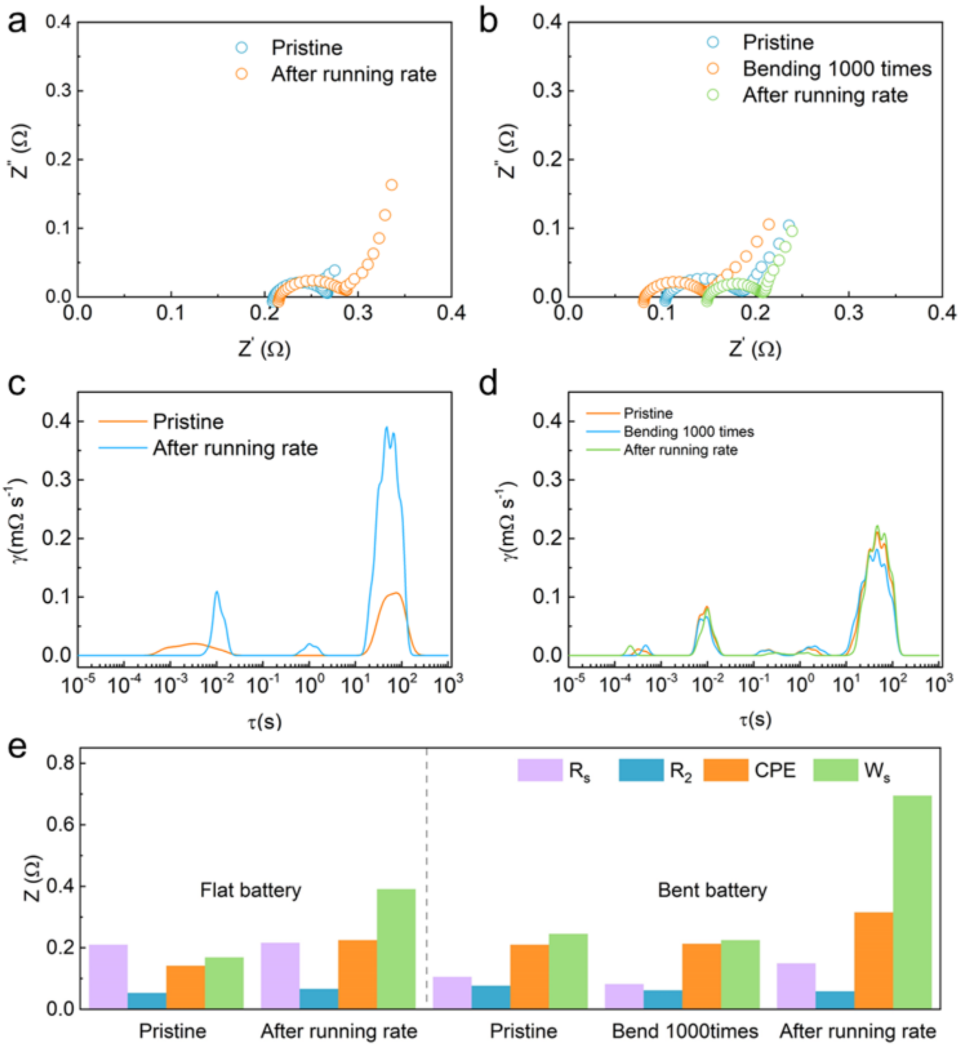

Table S1. The fitting data from Nyquist plots of the EIS spectra.

Battery		Flat battery		Bent battery		
State		Pristine	After running rate	Pristine	After bending 1000 times	After running rate
$R_s(\Omega)$		0.21023	0.21625	0.10512	0.08187	0.14932
$R_2(\Omega)$		0.05351	0.06654	0.07658	0.06207	0.05846
CPE	CPE-T	0.14164	0.2249	0.21035	0.21319	0.31487
	CPE-P	0.82787	0.7781	0.76479	0.77349	0.73208
$W_s(\Omega)$		0.16877	0.3908	0.24559	0.22487	0.69484

Figure S10. The electrochemical performance of flexible battery. (a) The sepcific capacity of static battery and battery with different deformations. (b) The sepcific capacity of static battery and dynamic battery.

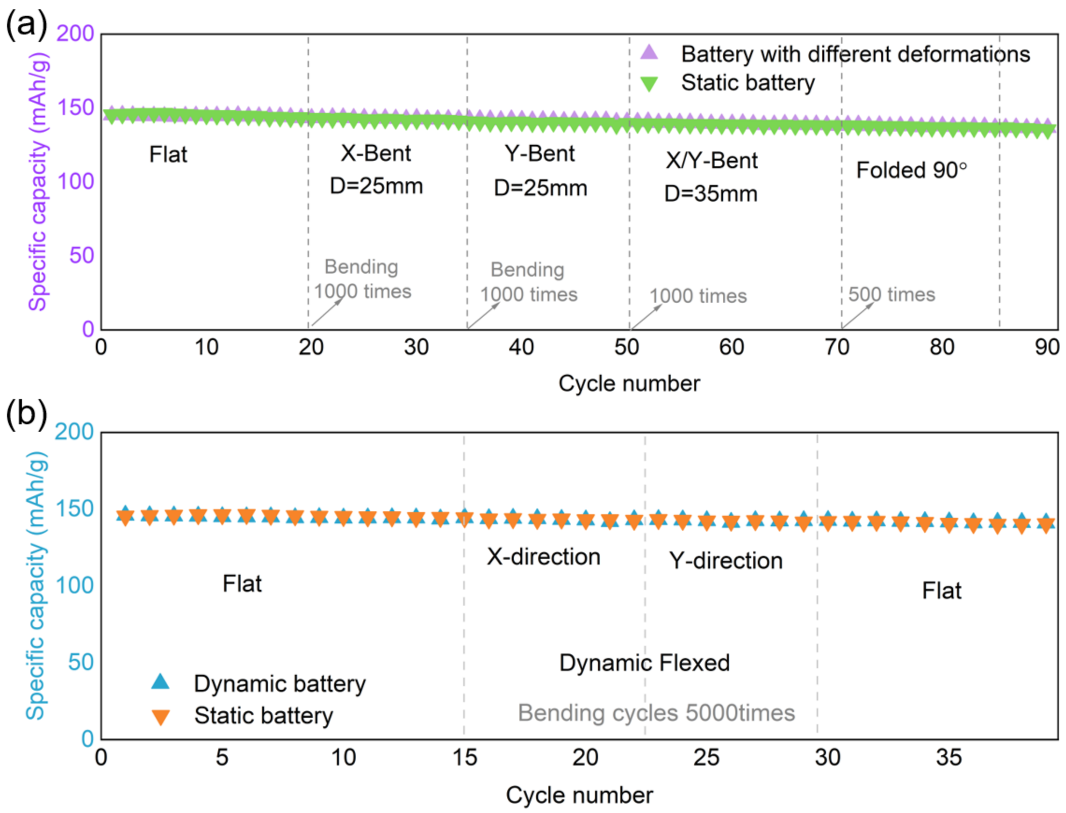
(a)
Specific capacity (mAh/g)
200
150
100
50
0
Battery with different deformations
Static battery
Flat
X-Bent
D=25mm
Y-Bent
D=25mm
X/Y-Bent
D=35mm
Folded 90°
Bending
1000 times
Bending
1000 times
1000 times
500 times
0
10
20
30
40
50
60
70
80
90
Cycle number
(b)
Specific capacity (mAh/g)
200
150
100
50
0
Flat
X-direction
Y-direction
Flat
Dynamic Flexed
Bending cycles 5000times
Dynamic battery
Static battery
0
5
10
15
20
25
30
35
Cycle number

Figure S11. Optical images of the snake-origami batteries under different mechanical deformational states: flat, bent and folded.

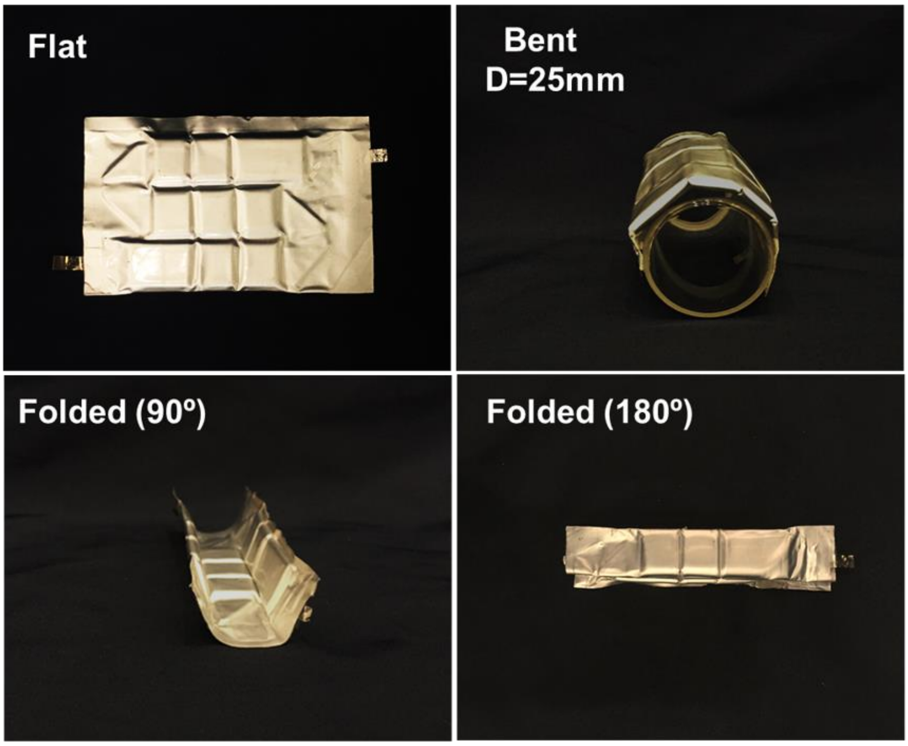

Figure S12. Distribution of relaxation time (DRT) results after treated the EIS spectra data with different mechanical deformation.

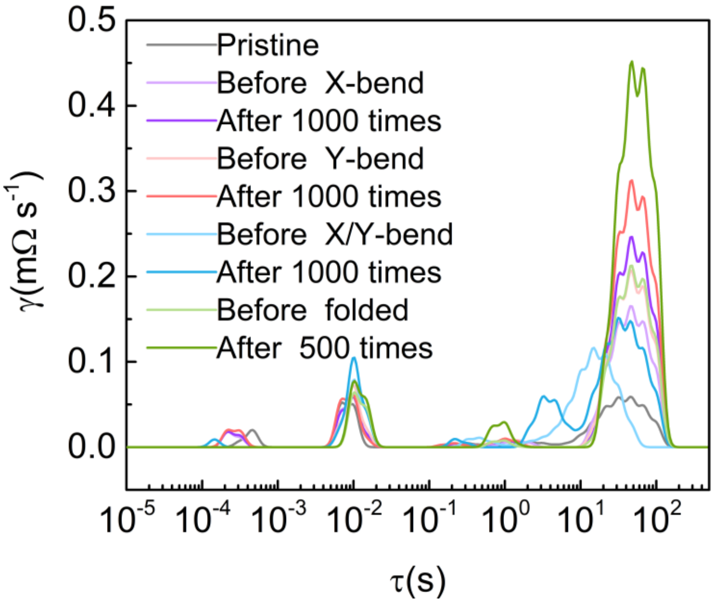

Table S2. The fitting data from Nyquist plots of the EIS spectra with different mechanical deformation.

Different state battery					
State	$R_s(\Omega)$	$R_2(\Omega)$	CPE		$W_s(\Omega)$
			CPE-T	CPE-P	
Pristine	0.07803	0.05018	0.17735	0.77487	0.09617
Before X-bend	0.09589	0.05016	0.24149	0.75855	0.17482
After X-bend 1000times	0.09468	0.04871	0.24201	0.7671	0.241
Before Y-bend	0.14988	0.04544	0.2135	0.86017	0.20777
After Y-bend 1000times	0.08360	0.05578	0.20684	0.77868	0.31012
Before X/Y-bend	0.2039	0.04120	0.21371	0.80382	0.16738
After X/Y-bend 1000times	0.22665	0.05208	0.13725	0.85648	0.24388
Before folded	0.25649	0.03511	0.19186	0.82912	0.20761

After folded 500 times	0.23712	0.04833	0.26944	0.77718	0.45299
Before twisted	0.29173	0.04756	0.17402	0.84057	0.28262
After twisted 1000times	0.30449	0.05245	0.23432	0.78043	0.27471

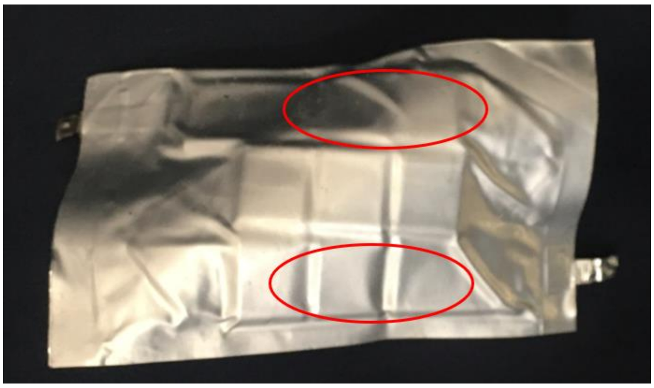

Figure S13. Optical images of the snake-origami batteries under twisted state.

Figure S14. The photographs of snake-origami batteries powering LED display screen under various bent state (a-d). The application for robot (e-f) and robot arm (g).

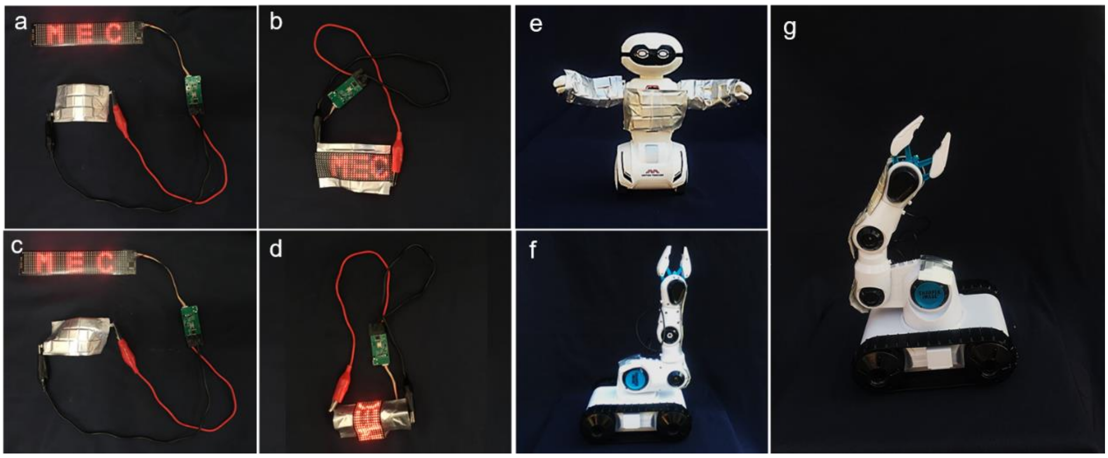

Figure S15. The charge–discharge curves of snake-origami batteries: (a) flat state and (b) dynamic loading state.

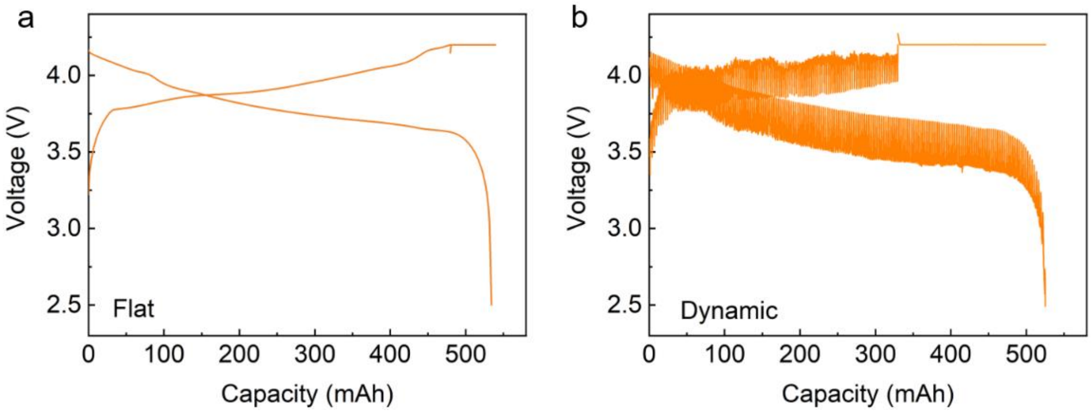

Table S3. The comparison of previous reported flexible batteries with snake-origami batteries.

Layer	Type of flexible	Electrode materials	Specific capacity		Energy density		Mechanical performance	Capacity retention	Ref.
			mAh/g	mAh/cm^3	Wh/kg	Wh/L			
Single-layer battery	3D battery	$LiCoO_2$/ Graphite	18.82	92.10	71.53	350	Single bend	≈80% after 4000 times of flexing	[21]
	Wave-like battery	$LiCoO_2$/ Graphite	14.43	11.08	56	43	Single bend	92.18% after 2000 bending cycles	[23]
	Thin battery	Commercial electrode	31.58	41.96	120	159.4	Single bend	99% after 1000 bending cycles	Panasonic
Multi-layers battery	Spine battery	$LiCoO_2$/ Graphite	34.43	63.9	130.15	242	Single bend/ Dynamic flexed	94.3% after different bending	[30]
	Zigzag battery	$LiCoO_2$/ Graphite	32.51	72.35	123.52	275	Single bend/ Dynamic flexed	96% after vary harsh deformations	[31]

	Wave-like battery	$LiCoO_2$/ Graphite	25	19.84	117.17	73.41	Single bend	95% after 5000 bending cycles	AEC
	Planar batteries	$LiCoO_2$/ Graphite	35.03	94.06	133.1	357.44	Bi-direction bend/ Dynamic flexed	94% after vary harsh deformations	This work

Video S1. The video of snake-origami batteries powering LED display screen under dynamic loading in X-direction.

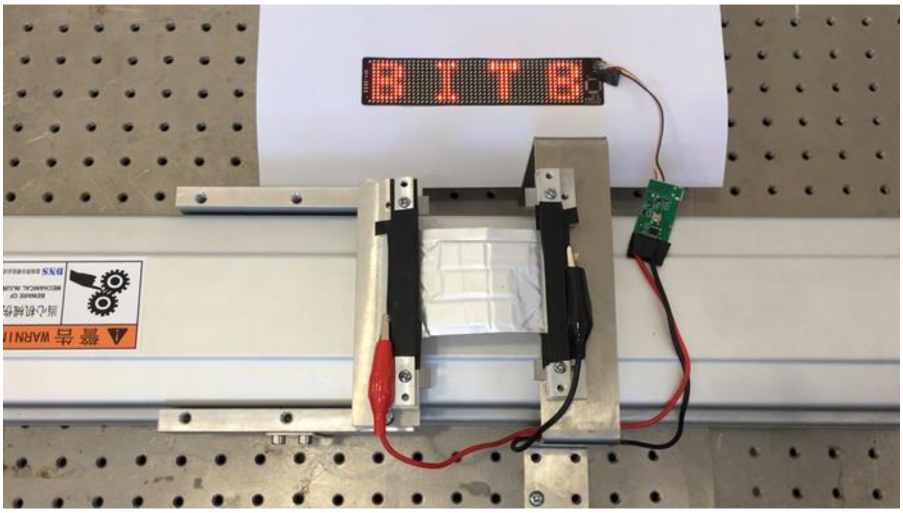

Video S2. The video of snake-origami batteries powering LED display screen under dynamic loading in Y-direction.

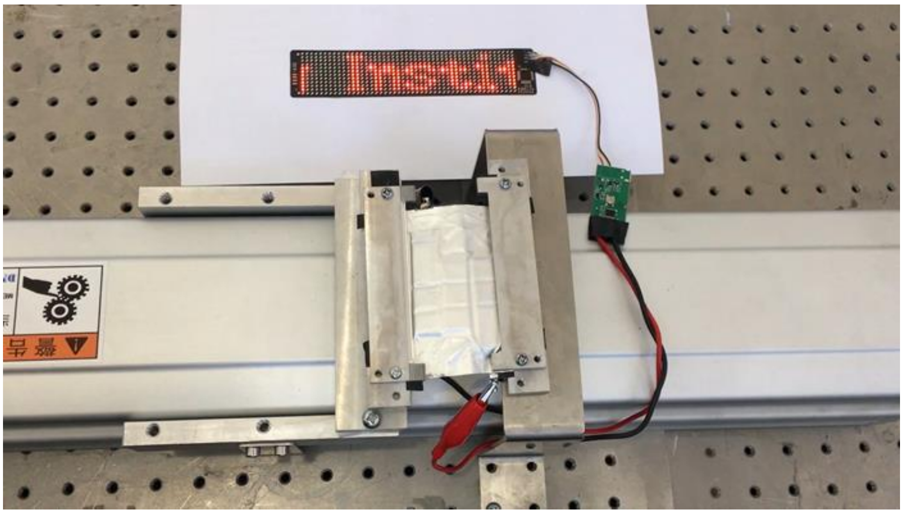

Video S3. The video of snake-origami batteries powering flexible LED display screen under dynamic loading, which the battery and LED display screen were tied together in X-direction.

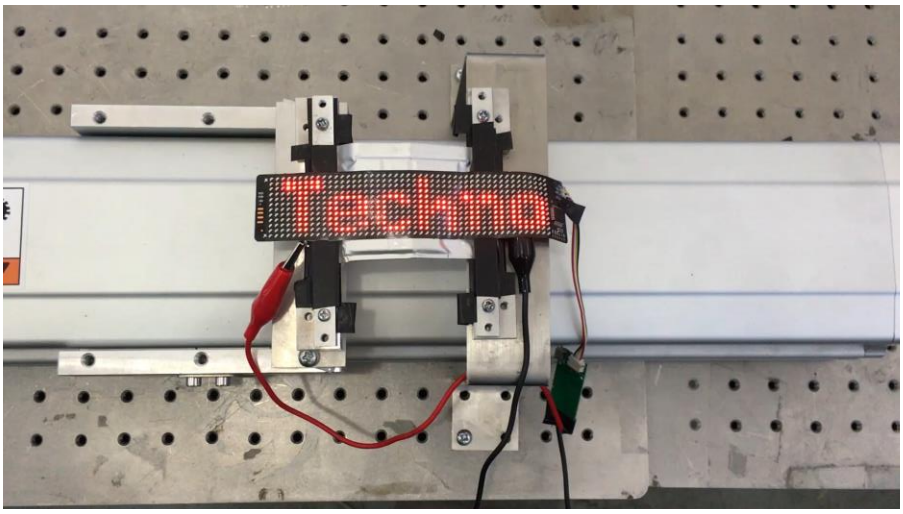

Video S4. The video of snake-origami batteries powering flexible LED display screen under dynamic loading, which the battery and LED display screen were tied together in Y-direction.

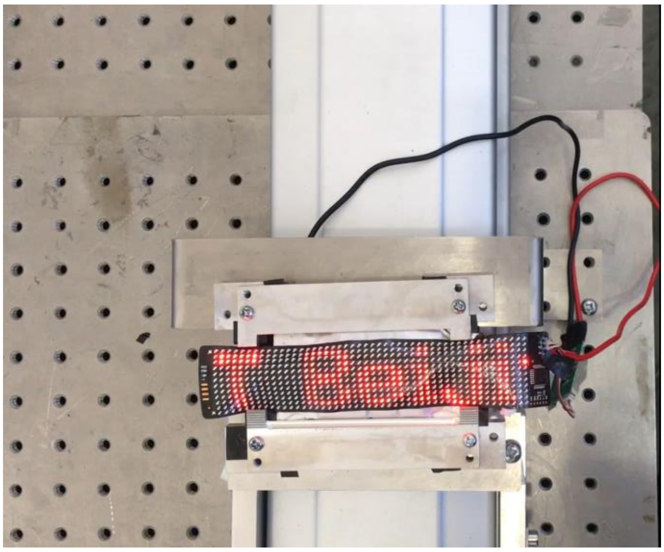

Supplement: Supplementary file 1 — Supporting Information [file ADVS-8-2101372-s003.pdf]
